# Latent Infection with *Leishmania donovani* in Highly Endemic Villages in Bihar, India

**DOI:** 10.1371/journal.pntd.0002053

**Published:** 2013-02-14

**Authors:** Epco Hasker, Sangeeta Kansal, Paritosh Malaviya, Kamlesh Gidwani, Albert Picado, Rudra Pratap Singh, Ankita Chourasia, Abhishek Kumar Singh, Ravi Shankar, Joris Menten, Mary Elizabeth Wilson, Marleen Boelaert, Shyam Sundar

**Affiliations:** 1 Institute of Tropical Medicine, Antwerp, Belgium; 2 Banaras Hindu University, Varanasi, India; 3 Barcelona Centre for International Health Research (CRESIB, Hospital Clínic-Universitat de Barcelona), Barcelona, Spain; 4 University of Iowa and the Veterans' Affairs Medical Center, Iowa City, Iowa, United States of America; The Faculty of Medicine, The Hebrew University of Jerusalem, Israel

## Abstract

**Introduction:**

Asymptomatic persons infected with the parasites causing visceral leishmaniasis (VL) usually outnumber clinically apparent cases by a ratio of 4–10 to 1. We describe patterns of markers of *Leishmania donovani* infection and clinical VL in relation to age in Bihar, India.

**Methods:**

We selected eleven villages highly endemic for *Leishmania donovani*. During a 1-year interval we conducted two house to house surveys during which we collected blood samples on filter paper from all consenting individuals aged 2 years and above. Samples were tested for anti-leishmania serology by Direct Agglutination Test (DAT) and rK39 ELISA. Data collected during the surveys included information on episodes of clinical VL among study participants.

**Results:**

We enrolled 13,163 persons; 6.2% were reactive to DAT and 5.9% to rK39. Agreement between the tests was weak (kappa = 0.30). Among those who were negative on both tests at baseline, 3.6% had converted to sero-positive on either of the two tests one year later. Proportions of sero-positives and sero-converters increased steadily with age. Clinical VL occurred mainly among children and young adults (median age 19 years).

**Discussion:**

Although infection with L. *donovani* is assumed to be permanent, serological markers revert to negative. Most VL cases occur at younger ages, yet we observed a steady increase with age in the frequency of sero-positivity and sero-conversion. Our findings can be explained by a boosting effect upon repeated exposure to the parasite or by intermittent release of parasites in infected subjects from safe target cells. A certain proportion of sero-negative subjects could have been infected but below the threshold of antibody abundance for our serologic testing.

## Author Summary

In this study we assessed trends with age in the probability of being sero-positive or sero-converting for two serological markers of visceral leishmaniasis (VL) among asymptomatic residents of high incidence villages. As markers we used Direct Agglutination Test (DAT) and rK39 ELISA. We also compared titers among asymptomatic sero-positives with those of known recent VL cases among our study population. Infection with VL is assumed to be permanent, but sero-positivity is a temporary phenomenon. Though clinical VL was most common among children and young adults (median age 19 years), we observed a statistically significant increase with age in the probability of being sero-positive and in the probability of sero-converting. We also observed that the average antibody titers among asymptomatic sero-positives were much lower than those among recent clinical VL cases. The increase with age in the probability of being sero-positive but also in the probability of sero-conversion can be explained by subjects experiencing repeated episodes of sero-positivity. This could be due to a boosting effect upon re-exposure, or to internal release of parasites from safe target cells. The implication of our findings is that in VL endemic areas it will be difficult to reliably distinguish between infected and non-infected subjects.

## Introduction

Visceral leishmaniasis (VL) or kala-azar is a parasitic infectious disease that is fatal if left untreated. Two *Leishmania* species are causal agents of VL: *L. infantum* and *L. donovani*. The first is a zoonosis and is endemic in countries around the Mediterranean basin and in Latin-America. The second is assumed to be an anthroponosis and is endemic in East-Africa and the Indian subcontinent [1].

India, Nepal and Bangladesh face a very high burden of VL. The ecological conditions for transmission of VL are very favorable in the Gangetic plains [2]. The State of Bihar in North Eastern India contains the biggest focus of VL, and reports about half of the world's annual new cases. In 2005 India, Nepal and Bangladesh launched a joint VL elimination initiative with a target of bringing the incidence down to less than 1 case per 10,000 by 2015. The main strategy to achieve this was early diagnosis and treatment, along with vector control measures. Key assumptions underlying this elimination strategy are that the disease is indeed an anthroponosis and that active cases of VL and post-kala azar dermal leishmaniasis (PKDL) are the only reservoirs maintaining disease transmission. During the past decade these critical assumptions have been questioned, and there are claims that animal reservoirs may play a role in *L. donovani* transmission [3], [4]. More recently Stauch et al [5] pointed to the possible role that latent carriers of *L. donovani* infection could play in transmission, if they were infectious for sand flies. One xenodiagnosis experiment in Brazil failed to demonstrate the ability of subclinicallly infected individuals with *L. infantum* to transmit parasites to the sand fly vector [6]. Nonetheless, given the biological differences between *L. infantum* and *L. donovani*, the different host, vector and epidemiological factors and the potential clinical and public health implications, these conclusions cannot necessarily be generalized to *L. donovani*.

The concept of latent infection with the agents causing VL has been well demonstrated. Indeed many individuals in endemic regions for *L. infantum* or *L. donovani* test positive for immunological markers of infection, but lack present or past symptoms of disease. In 1959, Manson-Bahr showed subjects without any symptoms of VL that were positive in the Leishmanin Skin Test (LST) in endemic areas in Kenya [7]. The term “asymptomatic infection” was used for the first time in 1974 by Pampiglione et al. based on LST responses in a region endemic for *L. infantum* in Italy [8], and later by Badaro et al. in Brazil [9]. Evidence for latent infection with *Leishmania* spp. has recently been expanded beyond tests of immunological response. As such, *Leishmania* spp. DNA was detected by PCR in the peripheral blood of asymptomatic human carriers in Brazil [10] and in Nepal [11]; Le Fichoux et al. [12] cultured promastigotes of *L. infantum* from the buffy coat of 9 out of 76 asymptomatically infected blood donors in southern France.

Several prospective studies have documented the ratio of asymptomatic infection to clinical disease by estimating the number of incident sero-conversions to incident new VL cases due to *L. donovani*. Bern et al. [13] in Bangladesh used LST and rK39 as markers of infection and found a 4 to 1 ratio between incident infection and disease. Ostyn et al. [14] used the Direct Agglutination Test (DAT) to document a 9 to 1 ratio in Bihar, India. Although ratios may be skewed because some of these “asymptomatically infected” individuals could progress to disease, the majority do not [15].

Proper assessment of the outcome of asymptomatic infection with parasites causing VL requires the identification of incident infections, and follow-up study of cohorts of these persons [16]. Ostyn et al. [14] showed a ten-fold higher risk of developing symptomatic VL among incident sero-convertors in India and Nepal compared to sero-negative controls. Such studies have been hampered by the fact that the routinely used antibody detection tests have shown excellent performance in detecting clinical cases when used in combination with a clinical case definition, but these tests have never properly been validated for the identification of asymptomatically infected persons [17]. The cut offs for positive serological tests have been chosen to separate healthy from diseased individuals, but not to separate infected healthy individuals from uninfected healthy individuals. Studies that document a working definition of asymptomatic infection with *L. donovani* are therefore needed.

This manuscript describes the results of two rounds of sero-survey which were conducted with the purpose of identifying a cohort of recent sero-convertors in Bihar, India. We use the data from the two baseline surveys to explore patterns in VL sero-positivity and sero-conversion in relation to age. Such baseline information will set the stage for downstream investigations of the spectrum of infected humans that can possibly serve as a reservoir for this pathogenic protozoan.

## Materials and Methods

The study was performed in the context of a larger ongoing longitudinal study in a high VL incidence area of Muzaffarpur district, Bihar State, India. The study site is a rural area comprised of 50 villages with a total population of 85,333, in which 193 VL cases were reported over a 2 ½ year period (March 2007–December 2009). To investigate *L. donovani* transmission and validate markers of infection, we set up a cohort of recent seroconverters, i.e. persons who were negative in leishmania markers at baseline, but converted during the follow up. To do so, we worked in the subset of villages with the highest VL incidence within the study area. We selected 11 villages with a total population of 19,886 individuals above 2 years of age, from which 144 cases of VL had been reported since March 2007.

### Study design and case definitions

Two house to house surveys were conducted with a one year interval in between. The first survey took place between December 2009 and February 2010. All residents above 2 years of age who were present and gave their informed consent were enrolled in the study. Informed consent was obtained from parents or legal guardians of subjects under age 18. Among subjects consenting to participate, a capillary blood sample was collected by finger prick on Whatman 3 filter paper. Samples were dried, after which they were packed in sealed plastic envelopes with silica gel. Filters were stored at −20°C until further processing.

DAT and rK39 ELISA were applied to detect antibodies against *L. donovani* in the participants sera; tests were performed as detailed elsewhere by Khanal et al. [16]. DAT titers were determined using a kit from the Institute of Tropical Medicine (DAT/VL, ITM) according to the manufacturer's instructions. Briefly, eight serial two-fold dilutions starting from an initial 1 in 200 dilution of serum were made. End titers for samples not reactive at the first step were classified as <1∶200, those still reactive in the final dilution step were classified as >1∶25,600. The recommended threshold for a positive DAT indicating VL is 1∶3,200; using this cutoff Harith et al. [18] arrived at 100% sensitivity and 99.3% specificity in a VL endemic district in Kenya. Joshi at el [19] in Nepal found DAT at a cut off of 1∶800 to be 100% sensitive and 99.2% specific for detection of clinical VL. Davies and Mazloumi Gavgani [20] and Saha et al. [21] used a cut off of 1∶1,600 to detect sub clinical infection, which is also the titer we opted for in this study.

RK39-ELISA results were expressed as the subject optical density (OD) value divided by OD value of a positive control serum sample ×100, and called percentage point positivity (pp) of a positive control. The cutoff chosen to define rK39 positivity was calculated as the mean value for a healthy non-endemic control plus three standard deviations. A log transformation was used to compensate for skewed distribution. The resulting value was 14 percent of the OD of a positive control. When endemic controls were used, the value increased to 23 percent of the OD of a positive control. To validate the cutoffs chosen we conducted a receptor operator curve (ROC) analysis based on comparison with confirmed recent VL cases since March 2007. We determined the cutoff point with the highest Youden index, i.e. the point with the highest combined sensitivity and specificity [22].

Sensitivity analysis was performed with higher cutoff values for both DAT and rK39. For DAT we used 1∶3,200, the conventional cutoff used for diagnosis of clinical cases; for rK39 ELISA we used the mean among non-cases in our (endemic) study population plus 3 standard deviations, i.e. 23 pp.

The study was part of a larger study in which information on episodes of active VL in the period between March 2007 and the time of the second survey had been collected for all subjects; episodes reported had been verified by a study physician [23]. During the survey, subjects were asked whether or not they had suffered from VL before March 2007.

### Statistical analysis

Data were analyzed using Stata/IC V10.1 (Stata Corp., College Station Tx, USA). Agreement between DAT and rK39 on a binary scale was assessed using Cohen's kappa coefficient [24]. Kappa coefficients were interpreted following Landis and Koch [25]: 1.00–0.81 excellent, 0.80–0.61 good, 0.60–0.41 moderate, 0.40–0.21 weak and 0.20–0.00 negligible agreement.

To assess the trends with age for the different markers studied, the study population was subdivided into 8 age groups (2–9; 10–19; 20–29; 30–39; 40–49; 50–59; 60–69; and 70+). For each age group the proportion of sero-positives and the proportion of VL cases were determined with their 95% confidence interval. Statistical significance of trends observed over age groups was assessed by chi squared for trend analysis. Logistic regression models were used to explore the associations between age as independent variable and serological markers or active VL as dependent variables. For this purpose we included in the model age, age squared and age to the power of three and used a backward elimination procedure, probability for removal was set at <0.05. The probabilities of suffering VL, being sero-positive and sero-converting were plotted by age.

We also assessed by age group the amplitude of the response for DAT and rK39, i.e. the median DAT or rK39 titer among sero-positives. For this purpose, DAT results were expressed in titer steps ranging from 1 (<1∶200) to 9 (>1∶25,600), rK39 results were expressed as percentage points as explained earlier. To test for statistical significance of differences in the distribution of DAT or rK39 titers by age group we used a Kruskal-Wallis test.

To assess the association between time elapsed since diagnosis and DAT or rK39 titers among ex-VL cases we used a Kruskal-Wallis test and linear regression on log transformed titers for rK39.

### Ethical considerations

This study forms part of a larger study for which ethical clearance was obtained from the review committee of the U.S. National Institutes of Health (NIH), as well as Institutional Review Boards of the Institute of Medical Sciences, Banaras Hindu University, Varanasi, India, and the University of Iowa. The IRB at Banaras Hindu University is registered with the US National Institutes of Health. Data was anonymized. All subjects provided written informed consent; in case of illiterate subjects a thumb print plus a signature of an independent witness were used. For minors under the age of 18 informed consent was obtained from a parent or guardian.

## Results

**Figure 1:**
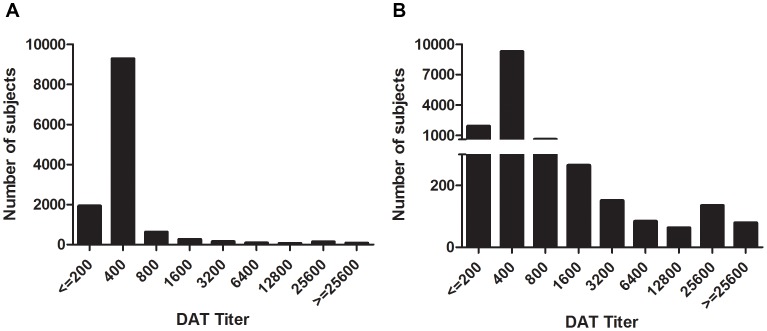
DAT titers at baseline. Serologic titers are graphed for the 12,634 subjects without prior or current VL, measured by the DAT test. Numbers of subjects with positive DAT tests at the indicated dilution are shown. The two graphs show the same data on different scales, so that both large and small titer peaks can be appreciated.

**Figure 2:**
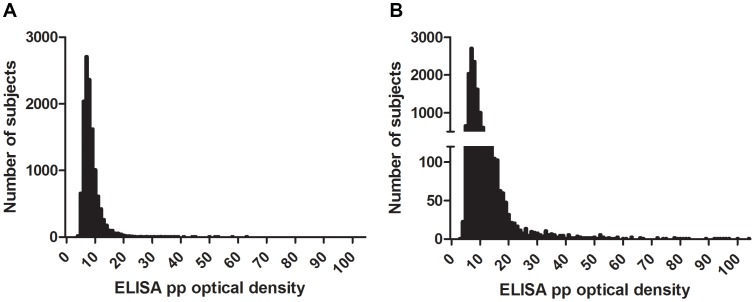
rK39 optical densities at baseline. Serologic titers are graphed for the 12,634 subjects without prior or current VL, measured by rK39 ELISA. The data are expressed as percentage of positive (pp), calculated as the (subject OD/positive control OD)×100 control for each subject. The left and the right figure show the same data on different scales, so that range of OD titers below 45 can be appreciated.

We enrolled 13,163 subjects, among whom were 118 individuals who had recently been diagnosed with VL (between March 2007 and December 2009), and an additional 411 individuals who reported a diagnosis of VL before March 2007. Individuals with former VL were all excluded from the first stage of the analysis. The DAT titers showed a peak of subjects around the 1∶400 dilution, and a much lower second peak at a much higher titer of 1∶25,600 (figure 1). The distribution of absorbance according to rK39 ELISA was skewed to the right with a steep peak at approximately 7 pp, but no second mode was observed at higher levels of optical density (figure 2).

### Seroprevalence

Based on the pre-specified cutoff criteria for differentiating positive from negative tests, 777 subjects (6.2%) were DAT positive (cutoff of 1∶1,600). Considering rK39 results with a cutoff chosen using serology form non-endemic controls (14 pp cutoff), 741 (5.9%) of the sera were rK39 positive. Agreement between the two assays was weak with a Kappa of 0.30 (95% CI 0.28–0.32). ROC analysis showed that for the rK39 ELISA, 14 percentage points was the value with the highest Youden index.

The alternative cutoff for rK39 ELISA of 23 pp resulted in 206 positive subjects (1.6%). Agreement with results of the DAT improved only marginally when using this higher cut off for rK39 in combination with a cutoff of 1∶3200 for DAT. (Kappa 0.31, 95% CI 0.30–0.33).

### Age dependent trends

Considering the trends in serologic response with age, we observed a steady increase with increasing age according to each of the serologic measures. The proportion of reactors according to DAT increased from 2.6% in the 2–9 year-old group to 15.9% in those aged 70 or older (table 1). Chi squared for trend was highly significant (p<0.001). Findings using rK39 were similar, although the proportion of reactors in the age group of 70 years and above was lower than that of the two preceding age groups. The confidence interval was wide, however, and the decline was not statistically significant. Overall the Chi squared for trend with age was highly significant (p<0.001).

**Table 1:** Trends in DAT and rK39 serologic responses by age group.

	Proportion (95% CI)		Odds ratios (95% CI)		
Age group	DAT positive	rK39 positive	DAT positive	rK39 positive	N
2–9	0.026(0.021–0.031)	0.021(0.016–0.025)	ref	ref	3,858
10–19	0.045(0.037–0.053)	0.045(0.037–0.053)	1.8(1.4–2.3)	2.2(1.7–3)	2,802
20–29	0.059(0.047–0.070)	0.061(0.049–0.073)	2.3(1.8–3.1)	3.1(2.3–4.1)	1,565
30–39	0.084(0.070–0.099)	0.072(0.059–0.085)	3.5(2.6–4.5)	3.7(2.7–4.9)	1,459
40–49	0.099(0.081–0.117)	0.094(0.076–0.112)	4.1(3.1–5.5)	4.9(3.6–6.7)	1,021
50–59	0.107(0.086–0.128)	0.118(0.096–0.140)	4.5(3.3–6.1)	6.3(4.7–8.6)	812
60–69	0.126(0.103–0.150)	0.138(0.114–0.163)	5.4(4.1–7.3)	7.6(5.6–10.2)	767
70+	0.159(0.119–0.199)	0.115(0.080–0.150)	7.1(5–10.2)	6.2(4.1–9.3)	321

**Figure 3:**
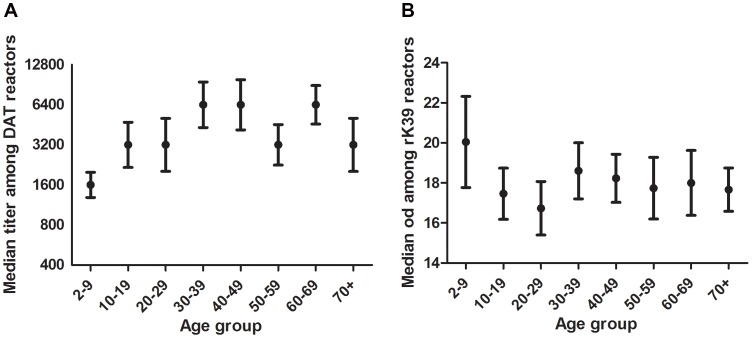
Median titer of positive DAT (left) or rK39 (right) tests by age. Among individuals who had a positive DAT response over or equal to 1∶1600 or a positive rK39 response over or equal to 14 pp, the median DAT titer (left) or rK39 pp (right) plus 95% confidence intervals are plotted for each age group.

We assessed whether the amplitude of the response among DAT or rK39 reactors was influenced by age (figure 3). The DAT titer appeared to increase in each age group up to age 40, and decrease among age groups over age 59 (figure 3 left). There was not a consistent pattern of rK39 titer (figure 3 right). Differences in median DAT titers between age groups observed among those above 40 years of age were not statistically significant (p = 0.92).

### Stability of serologic status

Between the first round sero-survey and the second sero-survey one year later, 2 VL cases were reported. Both were already rK39 positive (15.6 and 45.6 pp) and one was also DAT positive (end titer ≥25,600) at the time of the first survey round. During the second round survey, 252 DAT conversions and 145 rK39-ELISA conversions were documented. Among these, 16 persons had converted according to both tests. Among the DAT convertors, 19 were already rK39-ELISA positive during the first sero-survey, among the rK39-ELISA convertors 11 had already been DAT positive; thus in total there were 351 sero-negative individuals who seroconverted to DAT and/or rK39-ELISA over a year in our endemic study population. This constitutes 3.6% of the susceptible population of 9,873. The agreement between DAT and rK39 conversion was negligible, kappa = 0.071 (95% CI 0.052–0.090). The incidence of sero-conversion increased with age from 1.9% in the 2–9 years old to 7.0% in those aged 70 and above, chi squared for trend was highly significant (p<0.001) (table 2).

**Table 2:** Trends by age group in sero conversion to either DAT or rK39.

	Sero conversion		
Age group	Proportion (95% CI)	number	n
2–9	0.019(0.014–0.024)	65	3,415
10–19	0.027(0.021–0.034)	59	2,146
20–29	0.049(0.037–0.062)	54	1,093
30–39	0.041(0.029–0.052)	44	1,084
40–49	0.059(0.042–0.076)	45	765
50–59	0.063(0.044–0.083)	38	602
60–69	0.056(0.037–0.075)	31	555
70+	0.070(0.036–0.105)	15	213

Among 741 individuals who had a positive rK39 test identified in the first survey round, 626 were also sampled in the second round. Out of those, 372 (59%) had reverted back to sero-negative for rK39. Similarly, 777 individuals were initially DAT positives. Among these, 664 were sampled in the second survey and 216 of these (33%) had reverted back to DAT sero-negative.

**Figure 4:**
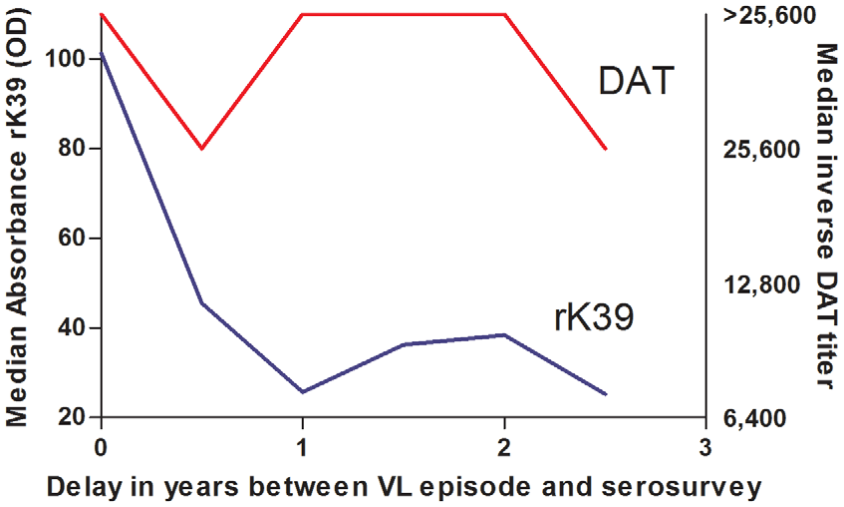
Median DAT and rK39 titers among 6-months cohorts of former VL patients in function of the time that elapsed since diagnosis (n = 118).

Included in the study population were 118 VL cases diagnosed between March 2007 and December 2009. Median age at time of diagnosis was 19 years. At the time of the sero-survey, 110 (93%) were still DAT positive and 96 (81%) were still rK39-ELISA positive; 116 (98%) were positive on either of the two assays. Average titers among ex-VL cases were clearly higher than those observed among subclinically infected subjects. The median DAT titer was >25,600 among ex-VL cases and 3,200 among subclinically infected; for rK39 the median titers were 33 pp and 18 pp respectively (figure 3 and 4). Whereas rK39 titers appeared to decline over time, DAT titers were fairly stable. The decline in rK39 titers was marginally statistically significant (p = 0.045).

Over the period of March 2007 till December 2009 (assuming a steady population) the highest VL incidence was among young adults in the 20–29 years age group; from this age onwards there was a steady decline, and there were no cases in persons above 70 years of age (table 3).

**Table 3:** Cumulative VL incidence by age group 2007–2009.

Age group	VL Incidence per 1,000 (95% CI)	OR (95% CI)	number	n
2–9	10.1(7.3–13.8)	ref	37	3,677
10–19	6.9(4.5–10.6)	0.7(0.4–1.2)	20	2,909
20–29	14.0(9.4–21.0)	1.4(0.8–2.4)	23	1,639
30–39	10.9(6.8–17.4)	1.1(0.6–1.9)	17	1,554
40–49	8.2(4.3–15.4)	0.8(0.4–1.7)	9	1,103
50–59	5.7(2.5–13.4)	0.6(0.2–1.4)	5	871
60–69	4.8(1.9–12.4)	0.5(0.2–1.3)	4	825
70+	0(0–11.4)	NA	0	332

**Figure 5:**
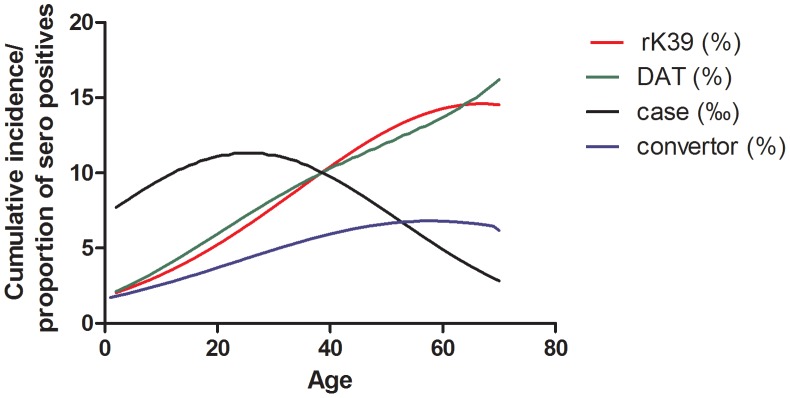
Probability of VL, sero conversion, and sero-prevalence by age group. Cumulative VL incidence over the 2½ year period preceding the first round sero-survey, probability of DAT and rK39 positivity during the first round sero-survey, and probability of sero-conversion on either DAT or rK39 during the 12-months interval between the first and second round survey have been plotted by age.

We modeled DAT and rK-39 positivity and the probability of active VL and sero-conversion against age as independent variable. The probability for being a VL case was highest at 25 years, with a steep decline from that age onwards. For sero-positivity and sero-conversion there was a steady increase with age, until 59 years for sero-conversion, until 67 years for rK39 positivity and beyond that age for DAT positivity (figure 5).

## Discussion

In this large sero-survey in highly VL endemic villages in Bihar, India, we found that at the time of our baseline survey 5.9% of the population aged 2 years and above who had never suffered from VL were rK39-positive; 6.2% were DAT-positive. There was limited overlap between both markers (kappa = 0.30), which is similar to what was observed in Ethiopia by Gadisa et al [26] and by Custodio et al [27].

Despite the observed increase with age, DAT and rK39 sero-positivity are temporary. Fifty nine percent of rK39 positives and 33% of DAT positives in the first survey round had lost their antibodies at the time of the second survey; similar observations were reported in other studies [28], [29], [30], [13], [14]. The discrepancy between the prevalence of the two serologic tests depends on the thresholds chosen but also on the relative rates of acquisition and loss of the markers. Among recent ex-VL cases we observed much higher titers than among subclinically infected but in this group too DAT titers appeared to be more stable than rK39 titers. Whereas the rK39 response appears to decrease over the time followed in this survey, the DAT response remained fairly stable over the 2½ year period of observation (figure 4). Notably, when the interval between disease episode and survey approached 2 ½ years, rK39 titers had fallen to levels similar to subclinical cases, whereas DAT titers remained much higher than subclinical subjects' titers (figures 3 and 4). The data suggest that the polyclonal response to the DAT total parasite antigen lingers longer than the monoclonal response to rK39. As such, one would predict the most likely time to find concordance between these serologic tests would be during acute infection, whether symptomatic or latent.

Although symptomatic VL was most common among young adults (age group 20–29 years) and became increasingly rare in the older age groups in our study, the frequency of both rK39- and DAT-positivity increased with age. We also observed an increase with age of the probability of sero-conversion on either DAT or rK39 between the first and second surveys.

An increase in leishmania sero-prevalence with age has been reported in other studies of *L. donovani* as well as *L. infantum* [31], [32], [33]. Although most active VL cases occur in younger age groups, infection with *Leishmania* spp. is assumed to persist for life [34]. Sero-positivity however is a time-limited phenomenon. The observation that nevertheless sero-prevalence and the frequency of sero-conversion increased with age could be explained by individuals experiencing repeated inoculations with infected sand flies, of which the early ones are more prone to lead to disease.

Hailu et al. [35] in Ethiopia report similar observations for LST, which reflects cellular immunity and is generally assumed to remain positive for many years or for life. The prevalence of LST positivity also increased with age, but individual subjects converted and reconverted in-between 7 rounds of sero-survey. Subjects were also tested with DAT and though prevalence levels were lower, DAT-positivity also fluctuated between surveys. Hailu et al. conclude that maintaining DAT or LST positivity requires continued exposure to *L. donovani*. Individuals may thus revert back to DAT negativity but rapidly reconvert to DAT positive on renewed exposure [35].

In our study population, the day-to-day intensity of exposure to *L. donovani* certainly varies during the year and between years [36], but at any given moment there is probably not much difference in the level of exposure between different age groups present in the villages. Older people may also have experienced periods of higher transmission in the past than younger individuals. However, the most likely explanation for the age-pattern we observed is that as people get older, their chances of ever having been infected increase. Upon renewed exposure they will more readily convert to a seropositive state in comparison to those that have never been infected before.

Another possible explanation for fluctuations in sero-positivity in asymptomatic individuals over time would be the occasional proliferation of parasites contained in safe target cells, i.e. cells that are not able to exert anti-parasite activities such as fibroblasts [37], [34]. Intermittent proliferation of parasites from such cells could lead to repetitive antigenic stimulation of the innate and adaptive immune system [38].

Assuming that, as also suggested by the observations of Hailu et al. [35], repeated sero-conversions are mainly due to repeated exposure, sero-conversion is probably an adequate measure of (re)infection. Sero-conversion has been used to evaluate public health interventions in Iran [39] and in India and Nepal where *L. donovani* sero-conversion and clinical VL results were correlated [40]. Infection is a necessary step in development of clinical VL, but it is not sufficient in itself [41]. Host factors also play a role, and individuals prone to develop disease are probably more likely to develop VL when first infected, at an early age.

Whichever the mechanism behind the increase in sero-prevalence and sero-conversion with age, the consequence is that in cross sectional surveys or in cohort studies that are too widely spaced, a substantial number of subclinically infected persons will turn out (false) negative on serology. This has implications for epidemiological studies and could have implications for control strategies as well. Though so far there has been little evidence for a role of asymptomatic infection in transmission of VL, more definitive measures of asymptomatic infection are needed before any firm conclusions can be drawn.

## Conclusion

In highly VL endemic villages in Bihar, India, substantial portions of the population react positive to DAT and/or rK39 suggesting they have asymptomatic infection. The proportion of seropositive individuals increased with increasing age. This pattern could be explained by a boosting effect in asymptomatically infected persons upon repeated exposure to the parasite or by intermittent internal release of parasites. Either mechanism could result in misclassification of asymptomatically infected persons in epidemiological surveys. Further follow-up is required to elucidate the significance of the sero-positive states according to both tests as markers of infection with *L. donovani*.

## Supporting Information

Checklist S1 STROBE checklist.
(DOC)
