# Correction: Latent Infection with *Leishmania donovani* in Highly Endemic Villages in Bihar, India

**DOI:** 10.1371/annotation/f096e293-9b24-46e6-bf1e-a18696611b4d

**Published:** 2013-04-09

**Authors:** Epco Hasker, Sangeeta Kansal, Paritosh Malaviya, Kamlesh Gidwani, Albert Picado, Rudra Pratap Singh, Ankita Chourasia, Abhishek Kumar Singh, Ravi Shankar, Joris Menten, Mary Edyth Wilson, Marleen Boelaert, Shyam Sundar

The eleventh author's name was incorrectly reported. The correct name is: Mary Edyth Wilson. 

