# Supplementary material for: Latent Infection with Leishmania donovani in Highly Endemic Villages in Bihar, India
Source: PLoS Negl Trop Dis. 2013 Feb 14;7(2):e2053. doi: 10.1371/journal.pntd.0002053 (PMC3573094; doi:10.1371/journal.pntd.0002053)
Supplement: Checklist S1 — STROBE checklist. (DOC) [file pntd.0002053.s001.doc]

STROBE Statement—checklist of items that should be included in reports of observational studies

|  | | Item No | Recommendation |
| --- | --- | --- | --- |
| **Title and abstract** | | 1 | √ (*a*) Two house to house surveys |
|  |  |  | √ (*b*) see abstract |
| Introduction | | | |
| Background/rationale | | 2 | √See introduction |
| Objectives | | 3 | √See introduction, last paragraph |
| Methods | | | |
| Study design | | 4 | √See ‘study design and case definition’ section |
| Setting | | 5 | √See ‘study design and case definition’ section |
| Participants | | 6 | √ (a)See ‘study design and case definition’ section |
|  |  |  | (b)Not applicable |
| Variables | | 7 | √See ‘study design and case definition’ section |
| Data sources/ measurement | | 8* | √ See ‘study design and case definition’ section |
| Bias | | 9 | Not relevant, we purposefully selected high incidence villages as described in the first paragraph of the ‘Materials and methods’ section. |
| Study size | | 10 | Not relevant, as mentioned in the final paragraph of the introduction the article describes the baseline results of a study in which a cohort of sero convertors will be identified. |
| Quantitative variables | | 11 | √ See ‘study design and case definition’ section |
| Statistical methods | | 12 | √ (*a*) See ‘statistical analysis’ section |
|  |  |  | √ (*b*) See ‘statistical analysis’ section |
|  |  |  | (*c*) Not applicable |
|  |  |  | (*d*) Not applicable |
|  |  |  | √ (*e*) See ‘study design and case definition’ section |
| Results | | | |
| Participants | 13* | √ (a) See ‘Results’ section, first paragraph | |
|  |  | (b) Not relevant | |
|  |  | (c) Not relevant | |
| Descriptive data | 14* | √ (a) See ‘Results’ section | |
|  |  | √ (b) In the section ‘stability of serologic status’ the numbers of participants who were followed up after one year have been indicated | |
|  |  | √ (c) In the section ‘stability of serologic status’ the numbers of participants who were followed up after one year have been indicated | |
| Outcome data | 15* | √ See ‘Results’ section | |
| Main results | 16 | (*a*) Not applicable | |
|  |  | √ (*b*) See table 1-3 | |
|  |  | Not relevant, absolute risks are provided (figure 5) | |
| Other analyses | 17 | √See ‘Results’ section | |
| Discussion | | | |
| Key results | 18 | √See paragraph 1 + 2 of ‘Discussion’ section | |
| Limitations | 19 | √ See ‘Conclusion’ section | |
| Interpretation | 20 | √ See ‘Conclusion’ section | |
| Generalisability | 21 | √ See paragraph 4 and 5 of ‘Discussion’ section | |
| Other information | | | |
| Funding | 22 | √ This work was supported by NIAID, NIH TMRC Grant No. 1P50AI074321;  R01 AI076233 from the US NIH; and a Merit Review and an OEF-OIF RFA grant from the Department of Veterans’ Affairs. The funders had no role in study design, data collection and analysis, decision to publish, or preparation of the manuscript. | |

*Give information separately for cases and controls in case-control studies and, if applicable, for exposed and unexposed groups in cohort and cross-sectional studies.

**Note:** An Explanation and Elaboration article discusses each checklist item and gives methodological background and published examples of transparent reporting. The STROBE checklist is best used in conjunction with this article (freely available on the Web sites of PLoS Medicine at http://www.plosmedicine.org/, Annals of Internal Medicine at http://www.annals.org/, and Epidemiology at http://www.epidem.com/). Information on the STROBE Initiative is available at www.strobe-statement.org.
